# Continuous Subcutaneous Versus Intestinal Levodopa Infusion for Parkinson's Disease: A Real‐World, Monocentric, Observational Study and Critical Review

**DOI:** 10.1002/mdc3.70557

**Published:** 2026-02-11

**Authors:** Johannes Hartig, Lennert Sitzmann, Doreen Hartenstein, Marion Labisch, Chi Wang Ip, Jens Volkmann, Christine Daniels

**Affiliations:** ^1^ Department of Neurology University Hospital Wuerzburg Wuerzburg Germany

**Keywords:** device‐assisted therapy, foslevodopa‐foscarbidopa, motor fluctuations, Parkinson's disease, pump‐based therapy

## Abstract

**Background:**

Subcutaneous foslevodopa‐foscarbidopa (SCFF) is a novel, non‐surgical dopaminergic infusion therapy for better controlling motor fluctuations in advanced Parkinson's disease (PD). However, there are scarce real‐world data on efficacy, adverse events and comparisons with other infusion strategies.

**Objectives:**

Here, we aimed to provide real‐world, observational data on treatment of advanced PD with SCFF infusion and compare and review its performance versus intestinal dopaminergic treatment strategies.

**Methods:**

We retrospectively collected monocentric data from patients with advanced PD treated with either SCFF (n = 58) or levodopa‐carbidopa(−entacapone) intestinal gel (n = 70). We extracted efficacy and adverse events in a real‐world setting and systematically reviewed the available literature for comparison.

**Results:**

One‐third of patients on SCFF withdrew from treatment within 4 weeks. Though generally deemed effective by both clinician and patient, there was a significant mismatch amid clinician (89%) and patient (74%) as per global clinical impression. Correspondingly, patients commonly withdrew due to preference rather than adverse events. Similar results were found for intestinal gel treated patients (89% vs. 70%). Comparison with intestinal levodopa & literature revealed that dose adjustments and adverse events in pump‐based therapies for PD are overall common, yet not systematically managed.

**Conclusion:**

Conclusively, our data suggest real‐world efficacy for SCFF in controlling motor fluctuations. However, there are significant dropout rates, side effects and patient‐clinician disagreement in global efficacy estimation. Comparison with intestinal infusion and literature revealed that pump‐based therapies lack structured management. We recommend the establishment of systematic guidelines for pump‐based therapies in advanced PD and provide a first troubleshooting algorithm for treating clinicians.

Fluctuations of motor and non‐motor symptoms are a defining feature of advanced Parkinson's disease (PD).^1^, ^2^ These fluctuations are, in part, intrinsically linked with levodopa absorption and dopamine metabolism.^3^ In particular, gastrointestinal transport problems and malabsorption of oral medication lead to increased (non‐)motor fluctuations, progressive disability, and thus, reduced quality of life. Two different, rarely complementary solutions are available to resolve fluctuations insufficiently addressed by oral medication optimization: deep brain stimulation (DBS), eg, of the subthalamic nucleus,^4^, ^5^ and pump‐based continuous infusion strategies.^6^, ^7^, ^8^ The latter include established therapeutic pillars for PD such as subcutaneous apomorphine, levodopa‐carbidopa intestinal gel infusion (LCIG, marketed as eg, Duodopa®),^9^ but also more recent approaches like levodopa‐entacapone‐carbidopa intestinal gel infusion (LECIG, marketed as eg, Lecigon®).^10^, ^11^ While showing comparable efficacy,^12^ both current pump‐based therapies and DBS are limited by individual patient characteristics, and specifically, invasiveness. Subcutaneous infusion on the other hand (including apomorphine^13^) offers the possibility of continuous drug delivery coupled with low invasiveness, ease‐of‐use, and thus low threshold for an individual treatment attempt.

Until recently, no approved forms of subcutaneous levodopa‐carbidopa existed, because the drug is difficult to bring into a stable, low‐volume hydrophilic solution. As of 2023, the prodrug foslevodopa‐foscarbidopa (currently marketed as Produodopa®) was approved for subcutaneous application based on results from a randomized, double‐blind, active‐controlled, phase‐3 trial for reducing motor fluctuations^14^ and a 12‐month open‐label phase‐3 study.^15^ The compound is applied subcutaneously and metabolized in situ by ubiquitous phosphatases into pharmacologically active levodopa and carbidopa. The stable levodopa/carbidopa plasma levels resulted in increased “on time” without dyskinesias, as well as reductions in “off time” at a solid risk–benefit profile, specifically for patients unable to undergo DBS surgery. Of note, the therapy is applied continuously over 24 h, in part probably due to the half‐life dominated by the metabolic conversion of the prodrug. This limits rapid adjustments of flow rate or bolus administrations, being well‐established features of other dopaminergic infusion therapies. Until now, there are scarce real‐world data on its day‐to‐day clinical use, specifically on complication rates, side effects, efficacy and comparability with other pump‐based strategies.

In this single expert center, real‐world, retrospective observational study and review, we thus collected and analyzed data from patients with advanced PD treated with subcutaneous foslevodopa‐foscarbidopa (n = 58) and intestinal levodopa‐carbidopa(−entacapone) (n = 70). We aimed to focus on real‐world tolerability, side effects, efficacy of subcutaneous foslevodopa‐foscarbidopa and comparison with established pump‐based therapies for advanced PD, including both literature and cohort data.

## Methods

### Data Extraction

Patient records (electronically available) of patients treated at the Department of Neurology, University Hospital Wuerzburg with either the new subcutaneous form of foslevodopa/foscarbidopa, LCIG, LECIG or a subcutaneous apomorphine pump were screened by using pump‐based therapy record keeping of our PD nurses. We then used patients’ whole clinical records and admission documentation to extract relevant variables and information into an excel data frame. This data frame was transferred into statistical analysis software.

### Systematic Literature Review

We used PubMed to screen the available literature for major studies conducted on the four forms of pump‐based therapies for PD to non‐systematically review the available data. We used the following search terms [((“Parkinson Disease”[Mesh]) OR (parkinson*)) AND (((((((((((subcutaneous foslevodopa) OR (subcutaneous foslevodopa‐foscarbidopa)) OR (levodopa infusion)) OR (intrajejunal levodopa)) OR (subcutaneous apomorphine)) OR (lecig)) OR (lcig)) OR (levodopa‐entacapone‐carbidopa intestinal gel)) OR (levodopa‐entacapone‐carbidopa)) OR (produodopa)) OR (duodopa))] and focused on the major phase‐3/approval trials, open label extensions and any real‐world data trials. We isolated clinical outcome measures and adverse events specifically.

We defined the following inclusion criteria for the systematic literature review: (1) Both retrospective and prospective clinical trials, RCTs, SRs and meta‐analyses, (2) sufficient reporting on patient outcomes and side effects, (3) English or German language, (4) all trials concerning advanced therapies in (idiopathic) Parkinson's disease by pumps (LCIG, LECIG, SCFF).

We defined the following exclusion criteria for the systematic literature review: (1) Letters to the editor, editorials, (2) insufficient reporting of outcomes and side effects.

The following filters were applied: “Clinical Study,” “Clinical Trial,” “Meta‐Analysis,” “Randomized Controlled Trial,” “Review,” “Systematic Review.”

### Global Clinical Impression Assessment

Global clinical impression (GCI) was used as an indicator for overall treatment response with regards to motor fluctuations and resultant improvement in quality of life. GCI was extracted based on full clinical records for each patient based on (a) the treating movement disorders specialists’ based treatment response in a binary manner (0 = no significant improvement of (motor) fluctuations [and associated quality of life improvement] or 1 = significant improvement of (motor) fluctuations [and associated quality of life improvement]) for status at discharge, (b) identically the patients’ own assessment.

### Levodopa Equivalent Dose Calculation

Individual initial dosage (diurnal flow rate) per SCFF patient was calculated according to levodopa equivalent dose (LEDD)^16^ of all oral dopaminergic medication (± adjunctives, ie, agonists, catechol‐O‐methyltransferase inhibitors, etc.; usually ~16 h treatment window).

### Statistical Analysis, Plotting and Data Reporting

Statistical analyses and plotting was conducted using GraphPad® Prism (Version 10.0). Assessment for normality was conducted by using Shapiro–Wilk tests and assessment of Q‐Q plots. Individual statistical testing was conducted on a group level via analysis of variance (ANOVA, one‐ or two‐way) or mixed‐effects models whenever possible and relevant, to assess factor and interaction effects. In case of non‐gaussian distribution of data, corresponding standard non‐parametric equivalents were used. For correlation analyses, we used Pearson's r or Spearman's r for parametric and non‐parametric data, respectively. Data plotting is described individually for each plot in the figure legends. Significance is annotated as * **≙** *p* < 0.05, ** **≙** *p* < 0.01, *** **≙** *p* < 0.001, **** **≙** *p* < 0.0001, if not stated otherwise. Source data are available from the corresponding authors upon reasonable request.

## Results

### Subcutaneous foslevodopa‐foscarbidopa improves fluctuations in a real‐life clinical setting, yet is associated with significant discontinuation rates

**Figure 1:**
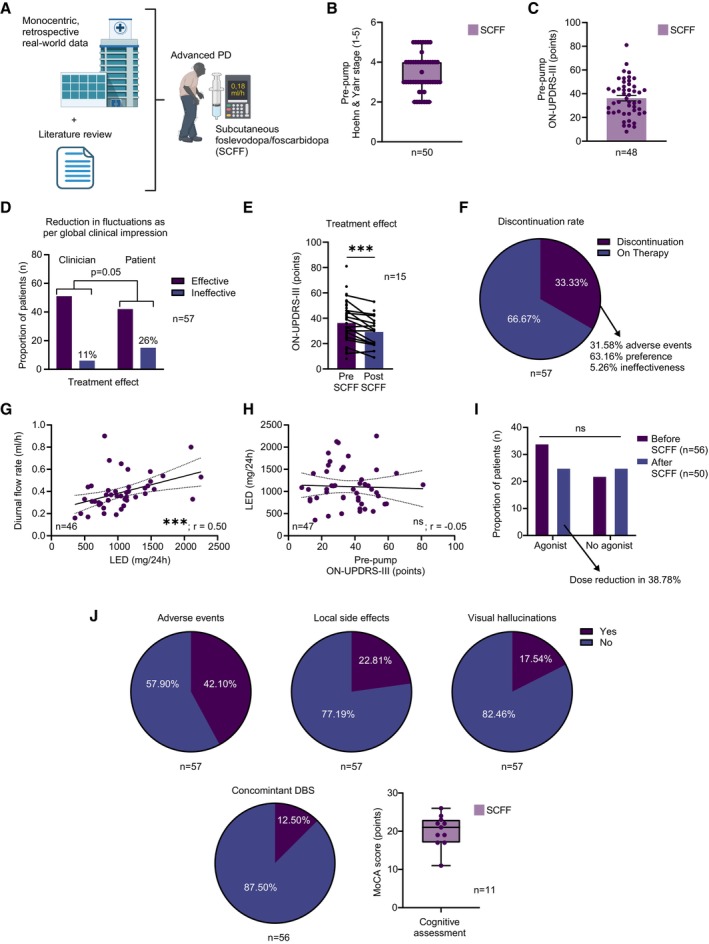
Clinical status, staging and outcomes of subcutaneous foslevodopa/foscarbidopa‐treated patients. (A) We retrospectively collected data from n = 58 patients treated with subcutaneous foslevodopa/foscarbidopa (SCFF) monocentrically. In addition, a comparison with available cohorts from literature was made.^14^, ^15^ (B) Per Hoehn & Yahr stage, advanced PD patients treated with SCFF had moderate‐to‐high disability (n = 50, median = 3, SD = 0.9605). (C) ON‐UPDRS‐III on oral levodopa (± adjunctives) before pump therapy (mean ± SEM = 36.19 ± 2.238) shows moderate‐to‐high clinical impairment of patients allocated to SCFF. (D) As per global clinical impression (GCI), both clinician and patient rated reduction in motor fluctuations (as per global clinical impression) by SCFF generally as effective. However, patients (~74%) less so than their clinicians (~89%) (Two‐sided Fisher's exact test, *p* = 0.0515). (E) ON‐UPDRS‐III on oral levodopa (± adjunctives) before pump and at last known follow‐up with SCFF shows a significant reduction (mean diff.: −6.0 points) in clinical impairment. (n = 15; two‐tailed paired *t*‐test, *t* = 4.255, *p* = 0.0008). (F) One‐third of patients withdrew from treatment after initiation of SCFF, yet 63.16% of those due to preference rather than ineffectiveness (5.26%) or adverse events (31.58%). (G) As expected, a highly significant correlation of levodopa equivalent dose (LEDD)/24 h and diurnal pump flow rate at discharge (Spearman's *r* = 0.5, *p* < 0.001) could be established. (H) There was no significant correlation of levodopa equivalent dose/24 h and pre‐SCFF ON‐UPDRS‐III on admission (Spearman's *r* = −0.05, *p* > 0.05). (I) The relative proportion of patients on agonist treatment did not differ after initiation of SCFF (Two‐sided Fisher's exact test, *p* = 0.3286), however dosage could be reduced in 38.78%. (J) Adverse events were overall common (42.10%), including local side effects (22.81%) and visual hallucinations (17.54%). Concomitant DBS was performed in 12.50% of patients, and a subsample of SCFF patients (n = 11) tested cognitively by Montreal cognitive assessment (MoCA) on average had no severe cognitive impairment (mean ± SEM = 20.09 ± 1.246).

Focusing on the subcutaneous foslevodopa‐foscarbidopa (SCFF) cohort first, we collected data from n = 58 patients (see Table 1 for demographics) from our movement disorders expert center with available data for downstream analysis and reviewed the current literature (Fig. 1A). We found that most patients in that group had moderate to high degrees of disability and corresponding clinical deficits on the Hoehn‐Yahr scale (H & Y scale, Fig. 1B, median: 3) and Unified Parkinson's Disease Rating Scale, Part 3 (ON‐UPDRS‐III on admission, Fig. 1C, mean: 36 points), respectively. We extracted the efficacy in reduction of motor fluctuations as a responder analysis based on the physician versus patient judgment of a clinically meaningful reduction in motor response fluctuations as the primary treatment goal from clinical records, assessing both the subjective judgment of the patient and treatment‐overseeing movement disorders specialist at discharge after pump therapy establishment (Fig. 1D).

**TABLE 1:** Demographic and clinical characteristics of patients treated with subcutaneous foslevodopa‐foscarbidopa (SCFF) and levodopa(−entacapone)‐carbidopa intestinal gel (L(E)CIG)

	Mean	SD	SEM	Median	95% CI of median (actual confidence level)	N
Subcutaneous foslevodopa‐foscarbidopa (SCFF)						
Age (in years)	71.4	8.464	1.111	72	95.21%	58
Age of onset (AOO)	56.96	9.948	1.366	56	97.30%	53
Hoehn & Yahr stage (1, 2, 3, 4, 5)	3.47	0.9605	0.1358	3	96.72%	50
Disease duration (in years)	13.26	6.23	0.8558	12	97.30%	53
OFF‐UPDRS‐III, pre‐pump (in points)	37.71	10.34	3.908	36	98.44%	7
ON‐UPDRS‐III, pre‐pump (in points)	36.19	15.51	2.238	34.5	97.07%	48
ON‐UPDRS‐III, post‐pump (first adm., in points)	29.22	12.14	2.861	30	96.91%	18
ON‐UPDRS‐III, post‐pump (last adm., in points)	14	2.828	2	14	50.00%	2
Levodopa equivalent dose (LEDD; mg/24 h)	1078	428.2	57.22	1050	95.60%	56
Pump rate (base rate; ml/h)	0.39	0.1541	0.02248	0.36	96.00%	47
Low pump rate (nightly; ml/h)	0.297	0.09211	0.01358	0.295	97.41%	46
Levodopa(−entacapone)‐carbidopa intestinal gel (L(E)CIG)						
Age (in years)	78.92	7.505	0.8444	79	95.78%	79
Age of onset (AOO)	59.32	9.495	1.169	60	96.44%	66
Hoehn & Yahr stage (1, 2, 3, 4, 5)	3.898	0.8076	0.101	4	96.72%	64
Disease duration (in years)	18.21	7.603	0.9358	16.5	96.44%	66
OFF‐UPDRS‐III, pre‐pump (in points)	55.5	5.568	2.784	56.5	87.50%	4
ON‐UPDRS‐III, pre‐pump (in points)	46.68	17.4	2.86	45	95.30%	37
ON‐UPDRS‐III, post‐pump (first adm., in points)	35.92	14.78	4.1	35	97.75%	13
ON‐UPDRS‐III, post‐pump (last adm., in points)	41.42	14.11	2.533	42	97.06%	31
Levodopa equivalent dose (LED; mg/24 h)	1015	424.3	52.63	921	95.36%	65
Pump rate (base rate; ml/h)	3.145	1.292	0.1578	3	95.02%	67
L(E)CIG‐Morning dose (ml)	8.325	2.673	0.3342	8	96.72%	64

Abbreviations: OFF‐UPDRS‐III, Unified Parkinson's disease rating scale‐part 3 in medication‐OFF; ON‐UDPRS‐III, Unified Parkinson's disease rating scale‐part 3 in medication‐ON.

While not the primary treatment goal, SCFF versus oral levodopa (± adjuvant drugs like dopamine agonists) also improved global motor function on UPDRS‐III in those patients that received SCFF (ON, exam at admission vs. discharge evaluation) significantly (Fig. 1E, mean difference: −6.067 points). Regarding improvement of ON‐time without significant dyskinesia, we found that both clinician and patient deemed the overall therapeutic effect on motor fluctuations as significantly beneficial (~74–89%). However, there was a discrepancy of ~15% of patients in whom the clinician saw solid clinical improvement, yet the patient did not. Importantly, we also found a high discontinuation rate (33.3%) within 4 weeks after initiating the therapy, of which most occurred due to patient preference (63.16%, Fig. 1F) rather than actual ineffectiveness (5.26%). Adverse events (AEs) caused 31.58% of treatment withdrawal.

As per approval studies, we determined the individual initial dosage (diurnal flow rate) according to levodopa equivalent dose (LEDD, see Methods section). Here, we found a significant correlation between the latter and final diurnal flow rate at discharge (Fig. 1G). However, we did not find a correlation of LEDD with ON‐UPDRS‐III before starting SCFF (Fig. 1H). While agonist treatment could be discontinued in some, apathy after agonist withdrawal prevented us from removing the agonist entirely in most patients, however dosage could be reduced in 38.78% (Fig. 1I). Any form of adverse event was quite common in the SCFF treated group (42.10%, Fig. 1J). Local side effects occurred in 22.81%, ranging from (more commonly occurring) infusion site reactions with erythema and nodules to fever, pain, significant swelling and even abscess. Mild (erythema; 7/13 patients) to moderate (swelling, pain, phlegmon; 4/13 patients) infusion site reactions were quite common in the SCFF group amongst those with local side effects, while severe ones (abscess, systemic inflammation/fever, etc.) were rather rare (2/13 patients). In our patients, the subcutaneous needles were exchanged every third day. If patients developed local side effects, the needles were exchanged more frequently, eg, every day. Since the initial phase‐3 trial^14^ reported a fraction of SCFF patients who experienced visual hallucinations, we aimed to check this in our real‐world cohort, too. Here we found that equally, ~18% of patients experienced visual hallucinations (Fig. 1J), partly in the context of delirium; however, few patients (5%) exhibited hyperactive delirium severe enough to cause significant aggression or agitation. Pump rates did require adjustment in about a fourth of patients, while 12.50% received concomitant DBS. For some patients (n = 11) cognitive assessment in the form of a Montreal Cognitive Assessment (MoCA) was available, which showed at least mild cognitive impairment in most SCFF designated patients (Fig. 1J).

### A Systematic Literature Review Shows Similarly Significant Discontinuation and Side Effect Rates

We then took these factors and systematically reviewed the available literature (see Table S1). In summary we here found the following: A blinded 12‐weeks phase‐3 trial for the pump used in our patients reported a similar ~35% dropout rate.^14^ A separate 52‐weeks open label phase‐3 trial^15^ by Aldred et al presented a comparable dropout rate of 43%. In our cohort, 33.33% of subjects discontinued the study due to AEs, with patient preference being the major reason. Frequently communicated factors were the size of the pump system and impairment of quality of life due to that. Further, patients were dissatisfied with magnitude of treatment effects. It should be noted that the subjects in the study by Aldred et al. had a lower H & Y stage (2.2 ± 0.7) and ON‐UPDRS‐III (23.5 ± 11.5 points), but similar LEDD (1064 ± 584.8 mg/24 h). Furthermore, infusion site events were amongst the most common AEs, as confirmed by studies by Soileau et al (72%)^14^ and Rosebraugh et al (40%).^17^ The most common local treatment complications included erythema, nodules and pain.

### Intestinal Levodopa in a Real‐Life Treatment Setting in Advanced PD Causes Similar Discontinuation Rates as Subcutaneous Foslevodopa/Foscarbidopa

**Figure 2:**
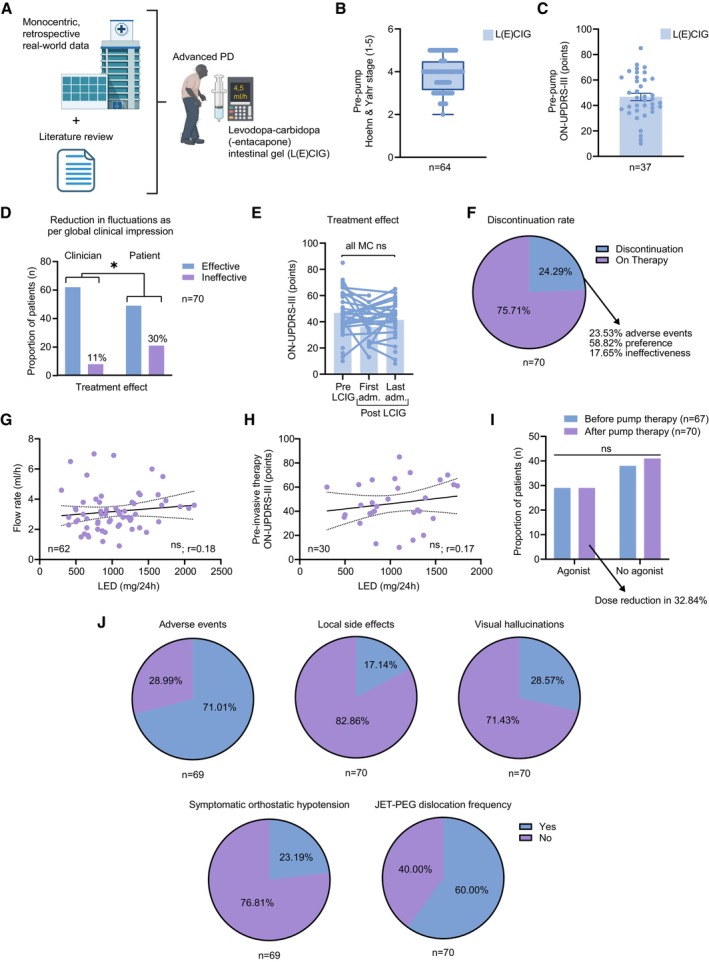
Clinical status, staging and outcomes of levodopa‐carbidopa intestinal gel‐treated patients. (A) We retrospectively collected data from n = 70 patients treated with levodopa‐carbidopa(−entacapone) intestinal gel (L(E)CIG) monocentrically. In addition, a comparison with available cohorts from literature was made. (B) Per Hoehn & Yahr stage, advanced PD patients treated with L(E)CIG had high disability (n = 64, median = 4, SD = 0.8076). (C) UPDRS‐III on oral levodopa (± adjunctives) before pump therapy (mean ± SEM = 46.68 ± 2.860) shows high clinical impairment of patients allocated to L(E)CIG. (D) As per global clinical impression (GCI), both clinician and patient‐rated reduction in motor fluctuations by L(E)CIG as effective. However, patients (70%) significantly less so than their clinicians (89%) (Two‐sided Fisher's exact test, *p* = 0.0115). (E) UPDRS‐III on oral/topical dopaminergic drugs before pump therapy as well as first and last known follow‐up. (Mixed‐effects model, treatment factor: *p* = 0.0714, n = 9 for pre versus first adm., n = 17 for pre versus last adm.; Dunnett's multiple comparisons tests: all *p* > 0.05). (F) Most patients stayed on L(E)CIG treatment once initiated, however there was a withdrawal rate of 24.29%, mostly due to preference (58.82%). (G) There was no significant correlation of levodopa equivalent dose/24 h and pump rate for LCIG cohort (Spearman's *r* = 0.18, *p* > 0.05). (H) There was no significant correlation of levodopa equivalent dose/24 h and pre‐pump ON‐UPDRS‐III (Pearson's *r* = 0.17, *p* > 0.05). (I) The relative proportion of patients on agonist treatment did not differ after initiation of LCIG (Two‐sided Fisher's exact test, *p* = 0.8549), however dosage could be reduced in 32.84%. (J) The L(E)CIG group experienced significant rates of adverse events, affecting >70% of patients. Visual hallucinations were more common (28.57%) than local side effects (17.14%). Adverse events specific to L(E)CIG treatment included dislocation of the jejunal tube through percutaneous gastrostomy (60%) and (aggravation of) symptomatic orthostatic hypotension (23.19%).

Concomitantly, we retrospectively evaluated the records of all available levodopa‐carbidopa intestinal gel (LCIG, Duodopa®) and levodopa‐carbidopa‐entacapone intestinal gel (LECIG, Lecigon®)‐treated patients from our center (n = 70) to achieve a robust comparison of clinical factors (see Table 1 for demographics) across major pump modalities (Fig. 2A). Again, we also systematically reviewed the available literature on L(E)CIG for comparison.

We found that these patients treated at our center had an even higher degree of disability before intestinal levodopa treatment at a median H & Y stage of 4 (Fig. 2B); correspondingly, clinical status was worse, too (Fig. 2C, mean ON‐UPDRS‐III on admission before pump therapy: 47 points). However, as with SCFF treatment, both clinician and patient generally deemed treatment with regards to reduction of motor fluctuations as effective as per global clinical impression (Fig. 2D). Yet again, there was a significant mismatch between the patient's and clinician's view. ON‐UPDRS‐III did not change significantly at both post‐implantation follow‐ups for the LCIG group either. And yet, discontinuation rates within the first year of LCIG treatment were significantly lower in the LCIG versus within 4 weeks of SCFF (~24 vs. 33%). Further, we found neither a significant correlation between LEDD and pump flow rate, nor of LEDD with ON‐UPDRS‐III pre‐implantation. Identical to the SCFF group though, clinicians were usually not able to withdraw patients from dopamine agonists, yet agonists could be reduced in 32.84%.

We then further dissected clinical characteristics in a real‐world setting and compared the pump modalities while extracting AEs and relevant clinical factors for L(E)CIG‐treated patients (Fig. 2J). We found that 71.01% of L(E)CIG versus 42.10% of SCFF‐treated patients experienced one or more adverse events (Fig. 1J and 2J). Importantly, almost 30% of L(E)CIG‐treated patients experienced visual hallucinations (Fig. 2J) versus 17.5% in the SCFF group (Fig. 1J) and significant aggression occurred in 10%. A similar but smaller fraction of L(E)CIG patients experienced a spectrum of local side effects (Fig. 2J, 17.14%). Patients on L(E)CIG commonly had mild (7/12 patients), yet only occasionally moderate (2/12 patients) or severe (3/12 patients) local side effects. Since L(E)CIG is known to cause or aggravate symptomatic orthostatic hypotension and dislocation of the jejunal tube through percutaneous gastrostomy (JET‐PEG) is seen frequently, we evaluated both former. Significantly, we found that more than half (60%) of L(E)CIG patients experienced at least one dislocation of their JET‐PEG (Fig. 2J) over totality of available follow‐up, causing a possibly significant amount of hospital readmissions and (temporary) clinical deterioration. About 23.19% of patients experienced symptomatic orthostatic hypotension. Only one patient in the L(E)CIG group was previously treated with DBS.

### Marked Side Effect Burden in Intestinal Levodopa Gel Treated Patients Revealed by Systematic Literature Review

Again, we aggregated the analyzed factors and systematically reviewed the literature (see Table S1). In summary, we here found the latter: most studies on LCIG^9^, ^18^, ^19^, ^20^, ^21^, ^22^ examined subjects with a lower baseline UPDRS‐III (18.1 to 28.8 points). LEDDs also varied from 861 to 1654 mg/24 h. The dropout rate of other studies differed greatly. In a study by Chaudhuri et al,^21^ 54.4% dropped out within 36 months, 27.2% of which were due to AEs. De Fabregues et al^19^ (37.8%, due to AEs 10.8%) and Buongiorno et al^18^ (38.8%, due to AEs 15.3%) showed similar figures. In Fernandez et al,^23^ only 23.2% (7.6% due to AEs) dropped out within 1 year and in Poewe et al,^20^ 26% (6% due to AEs) dropped out within 2 years. As with SCFF, local side effects such as erythema, infection, excessive granulation tissue or peritonitis were amongst the most common AEs reported in literature. In addition to infusion site events, neuropsychiatric symptoms were typical side effects. For example, 18% of subjects in one study^18^ experienced hallucinations. De Fabregues et al^19^ found that 35.1% of patients had hallucinations or psychotic events over a period of up to 10 years. However, Kovacs et al^22^ found only a 0.8–2.9% rate, with those subjects who received 24‐h pump therapy developing hallucinations more frequently (2.9%) versus 16‐h pump therapy (0.8%). In De Fabregues et al,^19^ 21.6% of the subjects had impulse control disorders. In the literature, dislocation or occlusion of the pump system, occurred at highly differing rates (22% dislocation, 12% blockage^9^ versus 56% dislocation, 57% blockage^23^). Yet, only 4% of 208 subjects in another study^20^ had dislocation, while 1% experienced occlusion of the system over a period of 24 months. Symptomatic orthostatic hypotension occurred in 27.3% of patients in one,^22^ yet in less than 5% in another two studies.^18^, ^21^ These results demonstrate that there are highly divergent rates of (specific) side effects in the available literature on major pump therapies, including from higher evidence trials. LECIG on the other hand is a relatively novel infusion treatment, too, which is why there is little data available, particularly regarding non‐motor side effects. However, a large international, prospective, observational study (ELEGANCE study, NCT05043103) is due to end in 2026 and will potentially shed light on the safety profile of LECIG. The results of two published studies^24^, ^25^ already provide preliminary information. The dropout rate was 25% and 33%, respectively, thus being relatively, but comparably high to the other pump‐based therapies. Viljaharju et al^25^ stated that 10% of discontinuations were due to AEs, which included hallucinations (3.3%) and delirium (3.3%). Öthman et al^24^ reported that 16.6% of participants dropped out due to AEs. The main reason was diarrhea; one subject developed hallucinations before JET‐PEG insertion. In total, 8.3% of the subjects in that study reported hallucinations on treatment. As with LCIG, dislocation and clogging also occur with LECIG. In Viljaharju et al,^25^ the pump dislocated in 23% and became clogged in 10% within 6 months. In Öthman et al, on the other hand, the pump dislocated and clogged in only 4% of cases, respectively, despite a longer observation period of 305 days.^24^

### Comparison of Intestinal Versus Subcutaneous Levodopa‐Treated Patients Highlights Shared Problems

**Figure 3:**
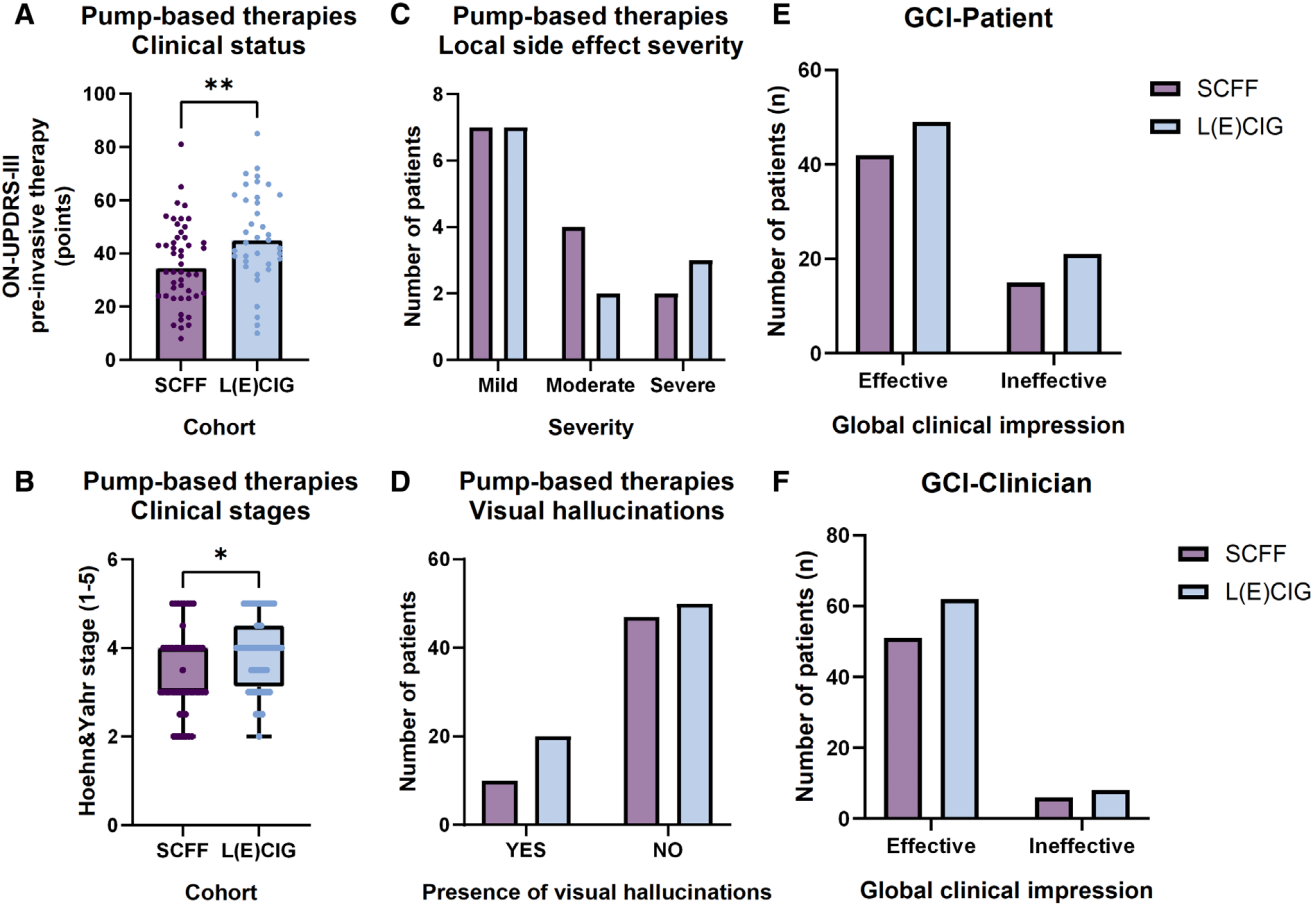
Outcome, side effects and status comparison between intestinal versus subcutaneous levodopa‐treated patients. (A) Levodopa‐carbidopa(−entacapone) intestinal gel (L(E)CIG) treated patients (n = 37) versus subcutaneous foslevodopa/foscarbidopa (SCFF) treated patients (n = 48) have worse motor scores (mean difference: 10.49 ± 3.631) pre‐pump implantation (unpaired *t*‐test, *t* = 2.888, *p* = 0.0051). (B) L(E)CIG patients (n = 64) have more advanced Parkinson's disease as per Hoehn‐and‐Yahr staging (actual difference: +1, Mann Whitney test, *p* = 0.0157) than SCFF‐treated patients (n = 50). (C) Relative number of patients affected by local side effects is not different between L(E)CIG and SCFF (Two‐sided Fisher's exact test, *p* = 0.7525). (D) Relative number of patients affected by visual hallucinations under therapy is not different between L(E)CIG and SCFF (Two‐sided Fisher's exact test, *p* = 0.2073). (E) Patient‐rated treatment effectiveness as per global clinical impression is not different between L(E)CIG and SCFF (Two‐sided Fisher's exact test, *p* = 0.6955). (F) Clinician‐rated treatment effectiveness as per global clinical impression is not different between L(E)CIG and SCFF (Two‐sided Fisher's exact test, *p* > 0.9999).

Finally, we compared intestinally versus subcutaneously treated patients with regards to clinical status, global clinical impression with regards to treatment efficacy and side effect burden. Here, we found that patients treated with intestinal levodopa were on average worse to begin with in both motor scoring and clinical staging (Fig. 3A,B). Interestingly, both local (Fig. 3C) and neuropsychiatric side effect (Fig. 3D) burden (visual hallucination presence), as well as overall effectiveness measures by patient (Fig. 3E) and clinician (Fig. 3F) were not very much different.

## Discussion

In this study, we provide real‐world, monocentric retrospective data on the recently approved subcutaneous foslevodopa‐foscarbidopa (SCFF) pump therapy (marketed as Produdopa®) for advanced Parkinson's disease (PD). Additionally, we collected data of PD patients treated with levodopa‐(entacapone)‐carbidopa intestinal gel for comparison and conducted a systematic literature review for an in‐depth relation with existing data on pump therapies for PD.

We found that the new pump modality was usually viewed as effective by both patient and caregiver in a real‐world setting, supporting other recent data.^26^ However, there was a discrepancy that is not yet well‐discussed in literature: Some patients did not think the therapy was effective, yet clinicians deemed so. Of note, patients’ dissatisfaction/preference was one major reason for discontinuation^27^; frequently communicated reasons were the impact of the size of the pump on quality of life and a dissatisfaction with magnitude of treatment effect. Withdrawal occurred at an overall rate of 33.33% in SCFF, which is in line with data from the 12‐month open‐label extension of the blinded 12‐weeks phase‐3 trial by Aldred et al.^15^ Another recently published, different subcutaneous levodopa‐carbidopa compound (ND0612) in a phase‐3 trial seems to work equally well,^28^ yet reported lower discontinuation rates.

In contrast, intestinal gel‐based therapy was discontinued less frequently (24.29%). This might be due to a lower degree of reversibility of L(E)CIG associated with increased burden in case of reversal, that is apparent to both patient and physician before treatment initiation. However, discontinuation rates in literature vary greatly for L(E)CIG.^18^, ^19^, ^21^, ^24^, ^25^ Dislocation of the jejunal tube (usually associated with significant clinical worsening and readmissions) occurred at least once in 60% of LCIG patients, which was more frequent than reported in literature, and yet, patients mostly remained on treatment. This indicates that while affected by major side effect burden, L(E)CIG patients on long‐term treatment usually remained on it, possibly because it represented a last‐line treatment, as indicated by average stage and severity of affection versus SCFF patients in our data.

In the available literature, all pump modalities, including also subcutaneous apomorphine, are generally effective and reasonably well‐tolerated,^18^, ^19^, ^20^, ^21^, ^22^, ^23^, ^29^, ^30^, ^31^, ^32^, ^33^ yet cause a non‐negligible amount of both neuropsychiatric and local side effects at ~5–25%. Ranging from mild local side effects to potentially highly disabling and troublesome hallucinations and full‐blown delirium or psychosis. Recent data on SCFF further highlight the critical nature of neuropsychiatric side effects and their potential link to cognitive impairment.^34^, ^35^ Reviewing the literature, it becomes apparent that pump‐associated adverse events, especially their structured management, has not received dedicated attention. That may in part reside in the fact that most pump therapies for PD are highly specialized, last‐line treatment modifications, and thus almost exclusively used in movement disorders expert centers. Second, focus is certainly on reduction of motor fluctuations rather than minimization of side effects. Of note, patients treated with intestinal versus subcutaneous levodopa were on average clinically worse to begin with but did not differ in effectiveness or local and neuropsychiatric side effect burden.

Evidently, this study has several important limitations. For one, it was conducted as a retrospective, observational design, which significantly limits the strength of evidence and conclusions to be drawn from it. A prospective, multicenter design would significantly improve our understanding of SCFF treatment due to the possibility of subgroup analyses with large sample sizes. Of note, post‐hoc analysis from a recent phase‐3 trial highlights preliminary evidence for effectiveness even in early disease course^36^ and a further real‐world trial is on its way (EARLY‐FOS, NCT06916507) with completion estimated in 2027.

Conclusively, SCFF treatment for advanced PD appears both overall effective and reasonably safe in a real‐world environment as a low‐invasive pump therapy. Yet, the ease of reversibility and discrepancy in patient versus physician rated effectiveness might contribute to relatively high discontinuation rates reported, including in literature, and thus economic burden. Moreover, adverse events in almost 50% of patients are not to be taken lightly, specifically being congruent with other pump modalities and available data. Neuropsychiatric (eg, hallucinations, delirium, psychosis) and severe local side effects might be specifically problematic—for the former of which predictive data would be highly valuable in a clinical setting. We hence advocate to thoroughly screen patient eligibility, focus on patient education and manage treatment expectations and complications well, thereby potentially improving pump therapies generally. Therein, it appears especially problematic that no structured management (guidelines) for pump therapies and associated adverse events exist so far, which we recommend being implemented across movement disorder centers internationally. We have provided a first troubleshooting algorithm for (movement disorder) neurologists treating patients with SCFF (see Fig. S1). It comprises a hitherto unvalidated expert proposal. The suggestions should thus be tested in prospectively designed studies, specifically, ineffectiveness, agonist withdrawal and side effect management.

## Author Roles

(1) Research project: A. Conception, B. Organization, C. Execution; (2) Statistical Analysis: A. Design, B. Execution, C. Review and Critique; (3) Manuscript: A. Writing of the first draft, B. Review and Critique.

J.H.: 1A, 1B, 1C, 2A, 2B, 3A.

L.S.: 1B, 1C, 3A.

D.H.: 1B.

M.L.: 1B.

C.W.I.: 1B, 3B.

J.V.: 1B, 3B.

C.D.: 1A, 1C, 3B.

## Disclosure

**Ethical Compliance Statement:** All experiments were conducted in accordance with the Declaration of Helsinki and approved via the local institutional review board (Ethics approval No. 2025‐83‐ka, University Hospital Wuerzburg, Lower Franconia, Bavaria, Germany). Informed patient consent was not necessary for this work. We confirm that we have read the Journal's position on issues involved in ethical publication and affirm that this work is consistent with those guidelines.

## Financial Disclosures and Conflicts of Interest

Author disclosures are available in the Supporting Information.

## Supporting information


**Figure S1** Troubleshooting flow diagram for problems on subcutaneous foslevodopa‐foscarbidopa. The flow diagram demonstrates our current approach to troubleshooting common problems under foslevodopa‐foscarbidopa pump therapy for two major problem areas: (A) Complications/side effects, (B) Dissatisfaction with treatment effect and possible solutions to those problems.

**TABLE S1** Studies on subcutaneous (fos)levodopa and intestinal levodopa identified by systematic review. Tables list study characteristics, evidence levels and treatment‐associated factors similarly investigated by our monocentric, retrospective data

**Data S1** Cois_all_concatenated.

## Data Availability

The data that support the findings of this study are available from the corresponding author upon reasonable request.
