# Supplementary figures and images for: Continuous Subcutaneous Versus Intestinal Levodopa Infusion for Parkinson's Disease: A Real‐World, Monocentric, Observational Study and Critical Review
Source: Mov Disord Clin Pract. 2026 Feb 11;13(7):1662–72. doi: 10.1002/mdc3.70557 (PMC13339380; doi:10.1002/mdc3.70557)

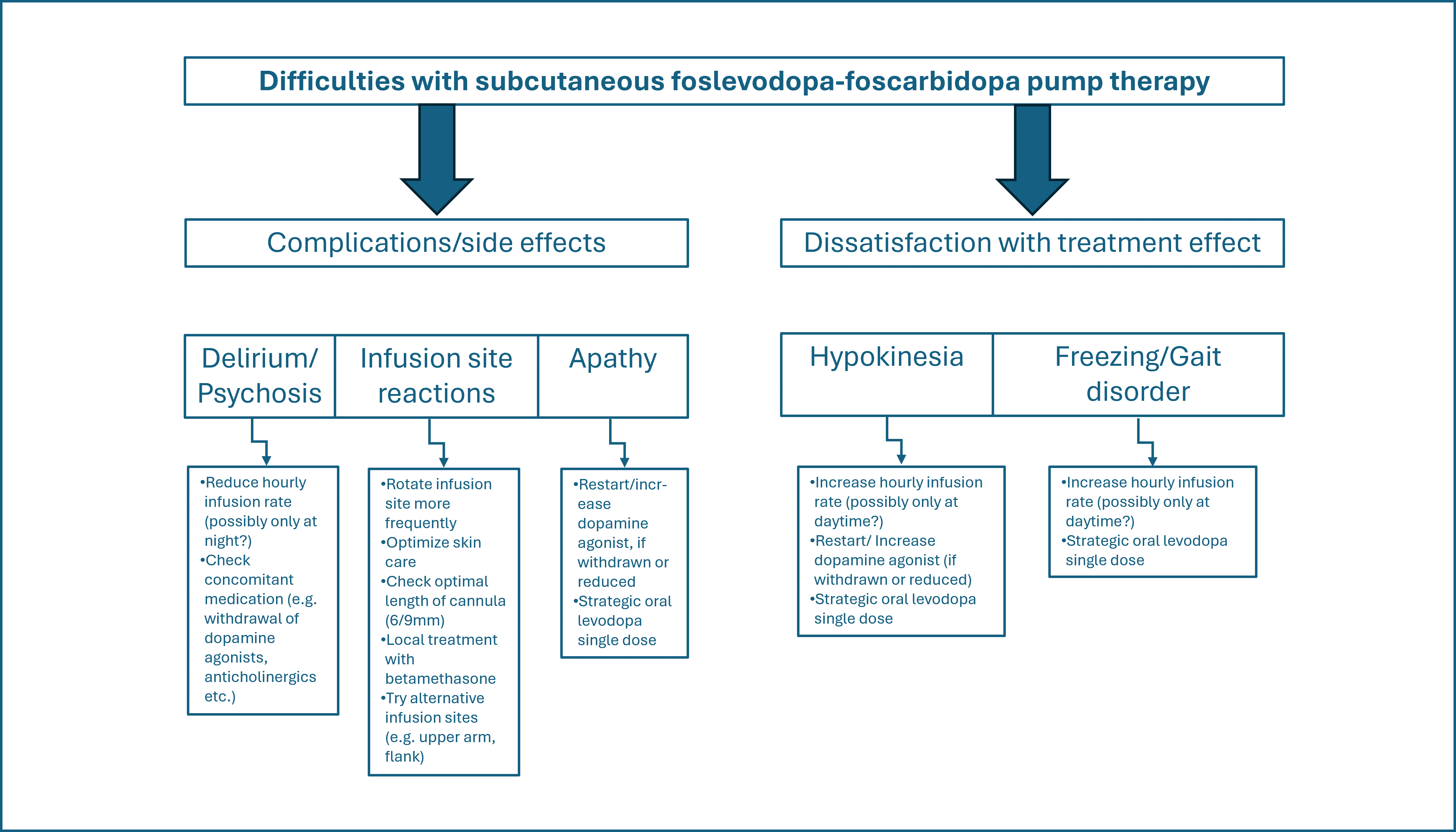

Supplement: Supplementary file 1 — Figure S1 Troubleshooting flow diagram for problems on subcutaneous foslevodopa‐foscarbidopa. The flow diagram demonstrates our current approach to troubleshooting common problems under foslevodopa‐foscarbidopa pump therapy for two major problem areas: (A) Complications/side effects, (B) Dissatisfaction with treatment effect and possible solutions to those problems. [file MDC3-13-1662-s002.png]
