# Supplementary material for: Continuous Subcutaneous Versus Intestinal Levodopa Infusion for Parkinson's Disease: A Real‐World, Monocentric, Observational Study and Critical Review
Source: Mov Disord Clin Pract. 2026 Feb 11;13(7):1662–72. doi: 10.1002/mdc3.70557 (PMC13339380; doi:10.1002/mdc3.70557)
Supplement: Supplementary file 2 — TABLE S1 Studies on subcutaneous (fos)levodopa and intestinal levodopa identified by systematic review. Tables list study characteristics, evidence levels and treatment‐associated factors similarly investigated by our monocentric, retrospective data [file MDC3-13-1662-s001.docx]

### **Supplementary Table 1**

# **Subcutaneous foslevodopa-foscarbidopa (Produodopa®)**

| **Study/DOI/**  **Duration/ Participants/ Oxford Level of Evidence** | **AEs** | **ISEs** | **Falls** | **Hallucinations** | **Delirium** | **Psychosis events** | **Discontinuation in total/reasons** | **Impulse control disorder** | **Hypotension** | **H&Y-ON** | **Pre-pump UPDRS-III** | **Post-pump UPDRS-III** | **LEDD** | **Agonist** | **Dis-location** | **Occlusion** |
| --- | --- | --- | --- | --- | --- | --- | --- | --- | --- | --- | --- | --- | --- | --- | --- | --- |
| **Soileau et al. 2022**  **(Phase 3)**  *10.1016/S1474-4422(22)00400-8*  *Week 12*  *n = 74*  *Level 2b (RCT, but <80% follow-up)* | 85% | -In total: 72%  -Erythema: 27%  -Pain: 26%  -Cellulitis: 19 %  -Edema: 12%  -No systemic complication | 8% | 15% | - | - | 22% | N/A | N/A | N/A | N/A | N/A | 1050 (800 – 1500) mg | N/A | N/A | N/A |
| **Aldred et al. 2023**  *10.1007/s40120-023-00533-1*  *54 weeks*  *n = 137*  *Level 2b* | 94,3% | -In total: 82%  -Erythema: 52%  -Nodule: 28,7%  -Pain:15,6%  -Abscess: 11,1% | 16,8% | 17,2% | - | 2,5% | -In total:43%  -AEs:22,9% (most: hallucinations 4,1%)  - Due lack of efficacy 4,5% | N/A | N/A | 2,2 (±0,7) | 23,5 (±11,5) | +1,8 (±12,9) | 1064,9 (±584.8) mg | N/A | N/A | N/A |
| **Rosebraugh et al. 2021**  **(Phase 1)**  *10.3233/JPD-212813*  *72 hours*  *n = 28*  *Level 2b* | 73,3% | -In total: 40%  -Dermatitis: 6,7%  -Erythema: 13,3%  -Nodule: 6,7%  -Pain: 13,3%  -Swelling: 6,7% | N/A | N/A | N/A | N/A | -Anxiety disorder (n=1) | 6,7% | 6,7% | N/A | N/A | N/A | -Group 1: ≤750 mg  -Group 2: 751-1250 mg  -Group 3: 1251-1750 mg  -Group 4: >1750 mg | N/A | N/A | N/A |

# **Subcutaneous levodopa-carbidopa (ND0612)**

| **Study/DOI/**  **Duration/ Participants/ Oxford Level of Evidence** | **AEs** | **ISEs** | **Falls** | **Hallucinations** | **Delirium** | **Discontinuation in total/reasons** | **Impulse control disorder** | **Hypotension** | **H&Y-ON** | **Pre-pump UPDRS-III** | **Post-pump UPDRS-III** | **LEDD** | **Agonist** | **Dislocation/ occlusion** |
| --- | --- | --- | --- | --- | --- | --- | --- | --- | --- | --- | --- | --- | --- | --- |
| **Espay et al. 2024**  *10.1016/ S1474-4422(24)00052-8*  *Week 6 (Open-label optimisation period)*  *n = 322*  *Week 12*  *(Double-blind double-dummy phase)*  *n = 128*  *Level 1b* | Week 6: 89%  Week 12: 80% | Week 6: 83%  Week 12: 57% | Week 6: 5%  Week 12: 7% | <2% | N/A | **Week 6** (open-label optimisation period)  -In total 19,6%  -Due to AEs 8,1% (infusion site events 5,9%)  -Due to inefficiency 2,2%  **Week 12**  -In total 6,25%  -Due to AEs 5,5% (infusion site events 2%; falls 2%) | Com-pulsive shopping (n =1) | N/A | ≤3 (inclusion criteria) | N/A | Week 12: +0,98 (±1,02) | 1079 (±487) mg | 0% | N/A |

# **Levodopa-carbidopa intestinal gel (LCIG)**

| **Study/DOI/**  **Duration/**  **Participants/**  **Oxford Level of Evidence** | **AEs** | **ISEs** | **Falls** | **Hallucin-ations** | **Delirium** | **Psychosis**  **events** | **Discontinuation in total/reasons** | **Impulse control disorder** | **Hypotension** | **H&Y- ON** | **Pre-pump UPDRS-III** | **Post-pump UPDRS-III** | **LEDD** | **Agonist** | **Dislocation** | **Occlusion** |
| --- | --- | --- | --- | --- | --- | --- | --- | --- | --- | --- | --- | --- | --- | --- | --- | --- |
| **Olanow et al. 2014**  *10.1016/S1474-*  *4422(13)70293-X*  *Week 12*  *n = 35*  *Level 1b* | 95% | -Erythema: 19%  -Wound infection: 11% | 11% | N/A | N/A | N/A | -In total: 7%  -Hallucination (n=1)  -Psychosis (n=1) | N/A | 14% | N/A | 18,1 | -1,5% | 1005,4 (± 373,6) mg | 59% | 22% | 14% |
| **Fernandez et al. 2014**  *10.1002/mds.26123*  *12 months*  *n= 324*  *Level 2b* | 92% | -Abdominal pain: 31,2%  -Granulation: 16,0%  -Wound infection: 15,4%  -Peritonitis: 2,8%  -Erythema: 13% | 15,1% | N/A | N/A | N/A | -In total: 23,2%  -AEs: 7,6% | N/A | N/A | N/A | 28,8 ± 13,7 |  | 1082,9 (± 582,1) mg | N/A | 1,5% | N/A |
| **Chaudhuri et al. 2023**  *10.3233/JPD-225105*  *36 months*  *n = 195*  *Level 2b* | 54,9% | N/A | 4,1% | N/A | N/A | N/A | -In total: 54.4%  -AEs: 27.2% | N/A | 2,19% | N/A | 27,6 ± 13,2 | +5,8 | 1529 mg | N/A | 2,6% | N/A |
| **Kovacs et al. 2022**  *10.1016/j.parkreldis.2022.08.002*  *>12 months*  *n = 401*  *Level 2b* | 27,3% | N/A | N/A | -24h-group: 2,9%  -16h-group: 0,8% | N/A | N/A | N/A | -24h-group: 2,9%  -16h-group: 0,5% | -24h-group: 20,0%  -16h-group: 20,2% | N/A | N/A | N/A | 1000 mg | N/A | 1,1% | -24h-group: 2,9%  -16h-group: 1,4% |
| **Fernandez et al. 2018**  *10.1002/mds.27338*  *52 weeks*  *n = 262*  *Level 2b* | 94% | -Erythema: 5%  -Abdominal pain: 10%  -Wound infection: 3% | 21% | N/A | N/A | N/A | -In total: 34%  -AEs: 24% | N/A | N/A | N/A | N/A | -4,77 | N/A | N/A | 56% | 57% |
| **Poewe et al. 2019**  *10.3233/JPD-191605*  *24 months*  *n = 208*  *Level 2b* | 39% | N/A | N/A | 1% | 1% | 1% | -In total: 26%  -AEs: 6% | N/A | N/A | N/A | 26,7 +- 13,4 | –5.5 ± 11.6 | 861,2 mg | Previous 81% | 4% | 1% |
| **Lopiano et al. 2019**  *10.1007/s00415-019-09337-6*  *­­­≈ 2,8 years*  *n = 145*  *Level 2b* | 27,6% | -Wound infection: 0,7%  -Peritonitis: 1,4% | N/A | 0,7% | N/A | N/A | -In total: 20,7%  - Due to  AEs 8,3% | N/A | N/A | N/A | N/A | N/A | N/A | During 29% | 4,8% | 7,5% |
| **De Fabregues et al. 2017**  *10.1002/brb3.758*  *≤ 10 years*  *n = 37*  *Level 2b* | N/A | -Peritonitis: 13,5%  -Granuloma: 37,8%  -Dermatitis: 32,4%  -Infection: 18,9% | N/A | (+ Psy-chosis) 35,1% | 29,7% (Con-fusion) | N/A | -In total:37,8%  -AEs:10,8% | 21,6% | 13,5% | N/A | 22.2 ± 8.4 | 21.1 ± 8.8 | N/A | N/A | 10,8% | 35,1% |
| **Palhagen et al. 2016**  *10.1016/j.parkreldis.2016.06.002*  *3 years*  *n = 77*  *Level 2b* | 80,6 – 100% | -Infection: 19,4%-27,8%  -Granulations: 11,1%-31,8% | 5,6%-22,2% | 16,7%-30,6% | N/A | N/A | -In total:26%  -AEs:17,9% | N/A | N/A | 2,0-2,5 | 24.4 (±11.0 | 22.0 (±9.7) | 1654–1366 mg | N/A | 27,8%-40,9% | 4,5%-13,9% |
| **Buongiorno et al. 2015**  *10.1016/j.parkreldis.2015.05.014*  *22 months - 48 months*  *n = 72*  *Level 2b* | N/A | -Wound infection: 6,9% | N/A | 18% | N/A | N/A | -In total:38,8%  -AEs:15,3% | N/A | 4,2% | N/A | (OFF: 40,7)  ON: 21,9 | (OFF: 39,9)  ON: 22,3 | N/A | N/A | 4,1% | 2,7% |

# **Levodopa-Entacapone-Carbidopa intestinal gel (LECIG)**

| **Study/DOI/**  **Duration/**  **Participants/**  **Oxford Level of Evidence** | **AEs** | **ISEs** | **Falls** | **Hallucinations** | **Delirium** | **Psychosis events** | **Discontinuation in total/reasons** | **Impulse control disorder** | **Hypo-**  **tension** | **H&Y- ON** | **Pre-pump UPDRS-III** | **Post-pump UPDRS-III** | **LEDD** | **Agonist** | **Dis-location** | **Occlusion** | **DBS** |
| --- | --- | --- | --- | --- | --- | --- | --- | --- | --- | --- | --- | --- | --- | --- | --- | --- | --- |
| **Viljaharju et al. 2024**  *10.1002/mdc3.13926*  *6 months*  *n = 30*  *Level 2b* | N/A | N/A | N/A | 3,3% | 3,3% | N/A | -In total: 33%  -AEs: 10%  (3,3% Delirium/  Hallucination) | N/A | N/A | 3,36 | N/A | N/A | 1230 mg | 44% | 23% | 10% | N/A |
| **Öthman et al. 2021**  *10.3390/jpm11040254*  *305 days*  *n = 24*  *Level 2b* | 29% | N/A | N/A | 8,3% | N/A | N/A | -In total: 25%  -AEs: 16,6% | N/A | N/A | N/A | N/A | N/A | N/A | 37,5% | 4% | 4% | 8,3% |

Abbreviations: AEs = Adverse events; ISE = infusion site events; H&Y-ON = Hoehn-and-Yahr scale rating in medication-ON; UDPRS-III = Unified Parkinson’s Disease Rating Scale-Item 3; LEDD = Levodopa equivalent dose; N/A = Not assessed
